# Immunity and neuroinflammation in early stages of life and epilepsy

**DOI:** 10.1111/epi.18361

**Published:** 2025-03-12

**Authors:** Angelica Vega García, María Leonor López‐Meraz, Marco I. González, Luisa Rocha, Jose Eduardo Peixoto‐Santos, Esper Abrão Cavalheiro

**Affiliations:** ^1^ Neurological Diseases Medical Research Unit Specialty Hospital, “Dr. Bernardo Sepúlveda”, National Medical Center “XXI, Century”, Mexican Social Security Institute (IMSS) Mexico City Mexico; ^2^ Laboratorio de Epilepsia Experimental Instituto de Investigaciones Cerebrales, Universidad Veracruzana Veracruz Mexico; ^3^ Department of Neurology University of California Davis School of Medicine Sacramento California USA; ^4^ Pharmacobiology Department Center for Research and Advanced Studies (Cinvestav) Mexico City Mexico; ^5^ Departamento de Neurologia e Neurocirurgia Escola Paulista de Medicina, Universidade Federal de São Paulo (Unifesp) São Paulo Brazil

**Keywords:** brain development, epilepsy, immune system, inflammation

## Abstract

The immune system is crucial for the correct brain development, and recent findings also point toward central control of immune response. As the immune system is not fully developed at birth, the early years become an important window for infections and for the development of epilepsy. Both central and even peripheral inflammation may impact brain function, promoting opening of the blood–brain/blood and cerebrospinal barriers and allowing entry of immune cells and cytokines, which in turn may affect neuron function and connections. The resident brain immune cells, microglia, besides providing protection, also affect neurons, myelination, and astrocyte function. They may, via the complement system, remove synapses, both physiologically and pathologically. After seizures during development, activated microglia releases proinflammatory molecules, which are detrimental for neurons, and inhibition of microglial activation shows promising antiepileptogenic effects. In addition to cytokines, seizures and excessive excitability stimulate calpain 2 expression, which can promote neuron loss and contribute to amplification of inflammatory responses via stimulation of proinflammatory cytokines. In summary, the immature immune system during postnatal early life may be an important target for the development of long‐desired antiepileptogenic drugs.

**Key points**

- New evidence points toward a complex interplay between brain and immune system during development.
- The immature immune system is permissive to inflammation, infection, and brain trauma.
- Besides brain protection, microglia shapes neuron–neuron interactions, neurogenesis, and neuron loss.
- Intervention on several pathways, such as the modulation of calpains, may prevent neuron loss and epileptogenesis.

## 1 INTRODUCTION

Recent discoveries regarding the interplay between the immune and nervous systems have significantly altered our understanding of the development and physiological functions of these important biological systems. Although we have learned that “pain, heat, redness, swelling, and loss of function” are central features of the inflammatory response, what would be enough to indicate the occurrence of simultaneous immune and nervous controls; these two systems have historically been considered almost completely independent. The central nervous system (CNS), for example, was traditionally considered an immune‐privileged site, a view that has evolved in recent decades.^1^

Advancements in our understanding of immune responses have revealed the existence of differential activation states among immune cells that can profoundly influence brain development and contribute to neurological diseases.^2^, ^3^ In addition, immune responses occurring outside the CNS can affect its functionality. For example, research indicates that increased peripheral inflammation can disrupt brain physiology, impairing hippocampal function and correlating with cognitive deficits in conditions such as schizophrenia.^4^, ^5^, ^6^

**FIGURE 1:**
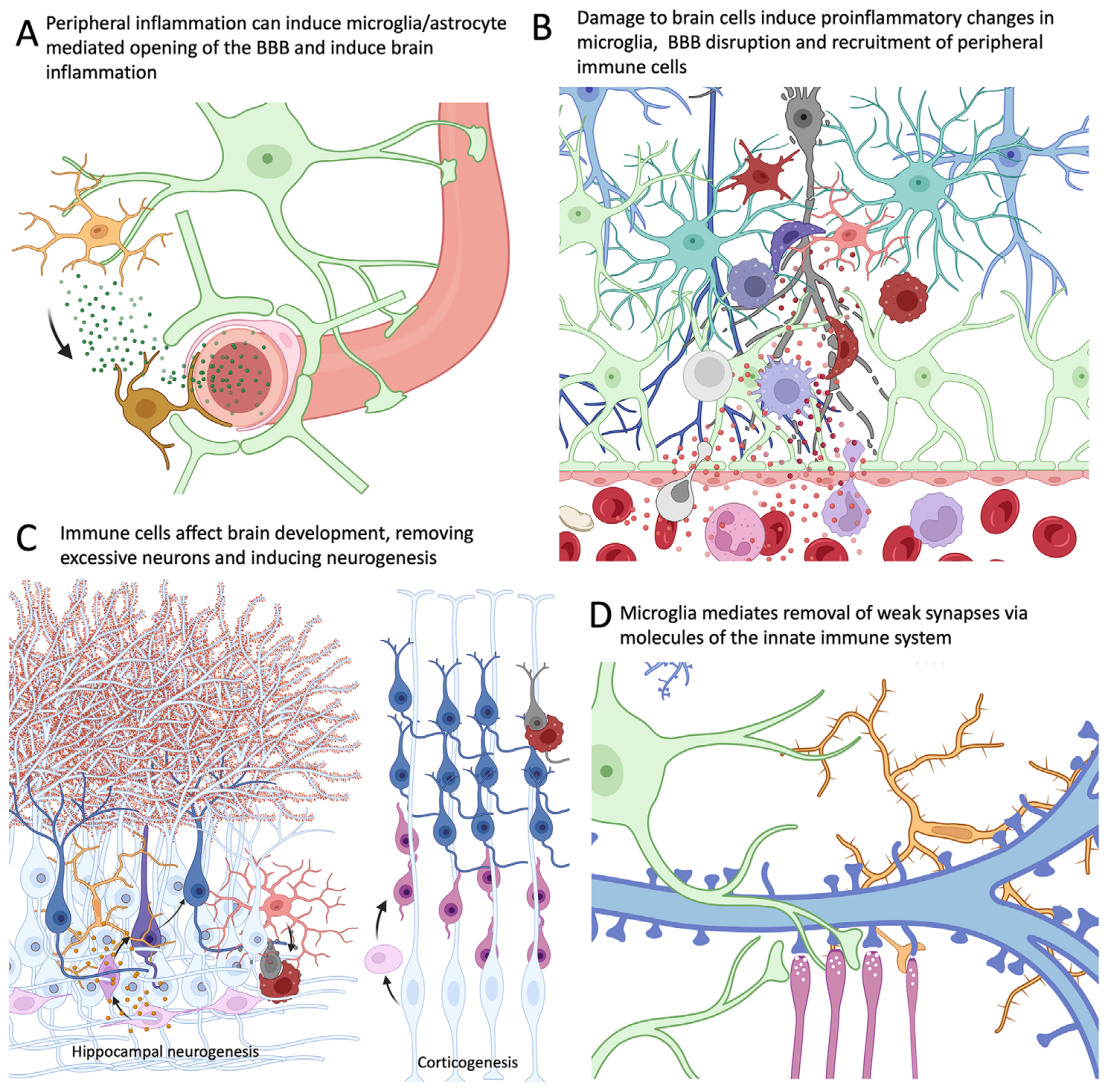
Crosstalk between the brain and immune system during development and injury. (A) Peripheral inflammation molecules (green dots) can induce blood–brain barrier (BBB) opening via microglia activation (yellow cells). Astrocytes are shown in green. (B) In addition, neuronal disfunction and neurodegeneration (gray cell) can also open BBB, with microglia (clear red cells) producing proinflammatory molecules that enter the bloodstream and induce lymphocyte (light gray cell) and macrophages (purple cell) migration. Both microglia (dark red cell) and blood macrophages (dark purple cell) can assume phagocyte morphology and remove cell debris. (C) Microglia also impact brain structure, removing (red cells) excessive neurons (dark gray cells) during corticogenesis and in the mature brain neurogenic niches. Microglia (yellow cell) can also release substances and induce neurogenesis. Stem cells are shown as pink cells, and radial glia is shown in light blue in the corticogenesis drawing. (D) Another crucial role for microglia (yellow cell) in brain development is the removal of weak synapses (thin pink axon and blue filiform dendrite) from neurons. Astrocytes (green cell) can also participate in synapse pruning. Created using BioRender (https://biorender.com).

These new findings suggest that the role of the immune system extends far beyond merely responding to threats against biological integrity (Figure 1).^7^ One of the most fascinating aspects of this relationship lies in the role the immune system has in the development and refinement of synaptic connections in the brain. To facilitate this process, the immune system deploys a variety of strategic mechanisms. Notably: (1) activation of CNS immune cells, the microglia, which identify and remove inactive or underutilized synaptic connections through a pruning process, optimizing neuronal circuit efficiency^3^; (2) activation of cytokines produced by immune cells that are crucial for neuronal growth and synaptogenesis^8^; and (3) interactions between microglia and immune receptors on neural cells, which can either facilitate or inhibit synaptogenesis.^9^

Conversely, the nervous system significantly influences the maturation and function of the immune system itself.^10^ During development, signals from the nervous system in response to stress or infection can impact the immune system's maturation, leading to lasting modifications in its normal functions. More recent findings indicate that the nervous system may also influence B‐cell development and antibody production, affecting antigen recognition and immune responses.^11^ Collectively, these scientific findings emphasize the reciprocal influence between the immune and nervous systems throughout development. They also highlight the potential consequences of any deviations, whether innate or acquired, that might disrupt this intricate interplay.

It has been known for some time now that specific brainstem cells could detect immune signals from the periphery and serve as master regulators of the body's inflammatory response.^12^ The exact location of these cells was not yet fully known, although the vagus nerve was already considered the ideal pathway for communication between the brain and peripheral structures.^13^ A recent and remarkable study was able to indicate that a group of neurons located in the caudal nucleus of the solitary tract, which receive direct input from neurons of the vagus ganglion, is able of transmitting anti‐inflammatory (expressing transient receptor potential ankyrin 1) and pro‐inflammatory (expressing calcitonin‐related polypeptide‐α) signals.^14^

Although the physiological role of this newly identified neuronal group during various phases of neural development is still being explored, understanding its function in the complex interaction between the nervous and immune systems is of considerable interest. As outlined above, communication between these systems occurs through a sophisticated network of specialized molecules, receptors on both neural and immune cells, and various neural pathways. Disruptions in this communication, whether due to genetic factors or environmental influences, can lead to a myriad of developmental disorders in the neural and immune systems.

In this sense, recent reports^15^, ^16^, ^17^, ^18^ suggest that a group of developmental epilepsies may arise from failures in this crucial communication between these two systems. To address this issue, this review explores relevant aspects that elucidate the underlying mechanisms through which impairments in neuroimmunological communication could facilitate the onset of epileptogenesis during development.

## 2 NEUROIMMUNOLOGY IN EARLY STAGES AND EPILEPSY

### 2.1 Immature innate and adaptive immune system during early stages

Host defense against pathogens in early development relies on the innate immune system as the first line of defense, and with development, the innate immunity interacts with components of the adaptive immune system. The different leukocytes develop and mature during fetal and postnatal life at different times, which explains the age‐dependent susceptibility to viral or bacterial infections.^19^, ^20^ For instance, the susceptibility to meningitis was associated with a combination of weaker intestinal and brain barriers, and a gut microbiota more permissive to streptococcus colonization in 5‐day‐old mice.^21^

Several studies have described the characteristics of immature cells of the innate immune system during development. Stimulation of immature monocytes with lipopolysaccharide ([LPS], a component of bacterial membranes) produces a lower increase in several proinflammatory cytokines when compared to adult monocytes.^22^ Another feature of the immature innate immune system response is the decreased expression of L‐selectin in neutrophils and of complement receptor 3 (CR3) in monocytes, which impacts their rotation and adhesion capacity.^23^ During human development, monocytes and dendritic cells (DCs) reach adult‐level expression of the specific cell surface receptors CD80 and HLA‐DR at only 3 months of age, whereas a response to LPS or other immunostimulant molecules is similar to adults only after 6–9 months of age.^24^ In addition, the expression of some cytokines after LPS stimuli is similar to that of adults after 3–6 months, whereas the expression of others remains lower even at 12 months.^24^ Other stimulants of the immune cells, such as oligonucleotides rich in CpG motifs, result in a level of expression of chemokines lower than that of adults even in cells of 12 month olds.^24^ Finally, the immature monocytes are less efficient in eliminating bacteria after phagocytosis.^25^ Overall, these studies reinforce the less‐effective response of monocytes in infants, which increases their susceptibility to infections.

The crosstalk between innate and adaptative immune cells is also less efficient in immature subjects. For instance, DCs from newborns present an attenuated expression of leukocyte antigens after LPS stimuli compared to adults. They also produce less interleukins and induce a lower Th1 response from T CD4 cells.^26^ Among lymphocytes, innate lymphoid cells are expressed in greater numbers during childhood, and are believed to have a role similar to that of T CD4 cells.^27^ In fact, these cells decline as Th2 CD4 cells increase during aging, reinforcing their complementary roles in defense.^27^ Natural killer (NK) cells, which increase during gestation reaching their maximum peak at birth, decrease in early life before increasing to the proportion found in adults. Moreover, the cells present a cytokine secretory profile instead of a cytotoxic profile, thus being less effective in removing infected cells.^28^ As for T CD8 and B cells, studies show that they can, in some settings, produce a response similar to that in adults.^29^ Lymphocytes also affect several aspects of brain development. T CD4 cells are crucial for microglia maturation and microglia‐dependent synapse plasticity, whereas B cells impact myelination via antibody‐dependent stimuli of oligodendrocyte precursor proliferation.^30^ These cells can also be detrimental for brain development, as maternal immune activation of T helper cells is linked to cortical malformations and reduced social interactions in the offspring and T cells activated by viral infection in early life increases lesions in the eperimental autoimmune encephalomyelitis model.^31^, ^32^ Specifically in children with epilepsy, although increased adaptative immunity was involved in already well‐known immune‐related etiologies such as Rasmussen's encephalitis, studies also revealed increased adaptative immune activity in non‐immune etiologies such as focal cortical dysplasia, tuberous sclerosis, and even in non‐lesional epileptogenic zones.^33^, ^34^, ^35^ Moreover, T‐cell density correlates with seizure frequency in several focal epilepsies, and T‐cell response was linked to postsurgical seizure control in tuberous sclerosis.^34^, ^36^ Animal models also reinforced the importance of adaptative immunity for focal epilepsy, including malformations of cortical development and hippocampal sclerosis.^37^, ^38^ In summary, adaptative immune cells play important roles in brain development and in epilepsy.

### 2.2 The blood–cerebrospinal barrier and blood–brain barrier in the postnatal stage

The blood–brain barrier (BBB) and blood–cerebrospinal barrier (BCB) are complex structures that serve as gatekeepers, protecting the CNS from pathogens.^1^ These barriers are functional from birth; however, complete maturation is achieved in adulthood, as evidenced by the increased activity‐dependent blood–oxygen level change and neural/glial vascular coupling in a functional magnetic resonance imaging (fMRI) study.^39^, ^40^ The BBB and BCB have important differences, with fenestrated capillaries and no tight junction at the choroid plexuses of BCB, which make the choroid plexus a main entry route for leukocytes, components of the immune system, and pathogens into the brain.^39^ Although inflammation‐driven changes in these barriers may help fighting microorganisms, such as increased lipocalin 2 expression, which reduces iron availability to bacteria and help controls bacterial growth, sustained peripheral inflammation is known to increase barrier permeability, thereby allowing immune cells and pathogens to enter into the brain.^41^, ^42^

Changes in BBB and BCB promoted by peripheral inflammation are proportional to stimuli and can be long‐lasting. For instance, although a single intraperitoneal LPS injection promotes changes in 46 genes of the choroid plexus that lasts up to 3 days,^43^ chronic stimulation changes the expression of 226 genes up to 15 days after the last injection.^44^ Thus faced with repeated LPS stimuli, the choroid plexus produces chemokines, complement molecules, and other molecules involved in the signaling of leukocyte invasion and activation of NK and T and B lymphocytes, as well as genes involved in synthesis of cytokines, thereby inducing brain inflammation.^44^ Although it is clear that chronic peripheral inflammation can cause inappropriate leukocyte recruitment into the brain in adults, these changes may also impact brain development after chronic inflammation in early life. For instance, repeated exposure to LPS alters the expression of genes encoding proteins involved in axonal guidance such as SLIT homolog 2 (SLIT2) in subventricular zone and choroid plexus, which acts as a chemorepellent on migrating neurons and extending axons.^45^ Another inflammation‐induced change with impact on development is related to increased secretin expression.^44^ This intestinal peptide hormone, which is expressed in the choroid plexus during development and in the dentate gyrus (DG) and other brain regions in adulthood, is involved in synaptic function.^44^ As secretin deficiency is associated with reduced dendritic spines and behavioral abnormalities in mice,^46^ increased release of secretin by the choroid plexus may lead to abnormal neuronal connections. Thus chronic peripheral inflammation can induce changes in the brain during development.

Astrocytes are crucial for BBB maturation and neurovascular coupling, attaching their endfeet to the BBB complex in early postnatal period.^47^ Several astrocyte proteins are important for BBB maturation, as deletion of astrocyte connexins impairs aquaporin 4 (AQP4) polarization, weakens BBB integrity, and produces edema.^47^ Moreover, the crosstalk between the components of BBB is crucial for their maturation, as shown by HMGB1 knockout mice.^48^ This protein, which can be released by macrophages and act in inflammatory situations, when inhibited in astrocytes also reduces connexin and AQP4 polarization around vessels and reduced astrocyte‐endothelial connections, and leads to endothelial abnormalities including vacuolization.^48^ As is well known, changes in AQP4 polarization are seen in hippocampal sclerosis, and animal models link this change to increased vulnerability to seizures.^49^, ^50^

### 2.3 Microglia and brain plasticity

Microglia take part in several neurodevelopmental processes, in addition to their role in physiological and pathological plasticity. They provide neurotrophic factors during neurodevelopment and can impair or induce neurogenesis via several mechanisms.^51^, ^52^ Microglia also seem important for angiogenesis, and facilitate axonal growth by modifying the extracellular matrix.^53^, ^54^ Microglia are so crucial for brain development that inhibition of nervous tissue colonization by these cells leads to ventricular enlargement, reduced cortex thickness, reduction of oligodendrocytes, some myelin deficits, and increased astrocyte population.^55^

One of the most striking roles of microglia in brain development is synapse pruning, which can be mediated by the immune system via on the activation of the classical component pathway. This mechanism is responsible for eliminating weak synapses, through complement 1q (C1q)–activated C3, which then binds to the synapses of the immature brain.^56^, ^57^ C3 binding to synapses target them for removal by microglia, the only resident cell expressing CR3 in the healthy brain, and is confirmed by studies with C1q, C3, and CR3 knockout mice.^56^, ^57^ The abovementioned mechanism is analogous to the function in the innate immune system, where C1q and/or C3 bind to cellular material, inducing its elimination by several different mechanisms, including phagocytic pathways. Astrocytes are also known to participate in the selective elimination of unnecessary synapses directly and via microglia stimulation.^58^, ^59^ The astrocyte‐induced microglial pruning relies on cytokines, as inactivation of IL‐33 production in astrocytes or its receptor IL1RL1 in microglia increases excitatory and inhibitory synapses density and reduces microglia‐dependent synapse elimination, thus showing a crosstalk between astrocytes and microglia for synapse pruning.^59^ Although the importance of pruning was classically related to schizophrenia, this mechanism can also promote hyperexcitability and epilepsy, as shown in a mice model with hyperactivation of the mechanistic target of rapamycin (mTOR) pathway in microglia (TSC1^Cx3cr1^ conditional knockout). These mice present increased synapse pruning without significant cytokine release, and develop spontaneous seizures early in life.^60^ However, a recent study showed that neurons and astrocytes also presented mTOR hyperactivation in the TSC1^Cx3cr1^ conditional knockout.^61^ Further studies are needed to clarify the role of pruning in epilepsy.

### 2.4 Microglia and status epilepticus in the immature brain

The classical view of microglia focused on their role in immune surveillance. However, in recent decades, this perspective has evolved, highlighting their crucial role in both the development and maturation of the CNS, as well as in modulating neuronal function.^62^, ^63^ One example of this is the response of microglia to neuronal hyperactivity, as seen in epilepsy. In fact, activated microglia are often seen in the epileptogenic zone of patients with epilepsy.^50^ In addition to the morphological changes microglia undergo during activation—transforming from ramified small‐body cells to amoeboid cells^64^—microglia also exhibit functional diversity. They can be categorized into two phenotypes: M1, which promotes inflammatory and neurotoxic effects, and M2, which plays a role in anti‐inflammatory and neuroprotective actions.^65^ In some neurodegenerative diseases, M1 microglia may shift to the M2 phenotype, a change that could mitigate harmful effects.^66^

The characterization of microglia in epilepsy is complex. Their response may vary based on factors such as the developmental stage during which the seizures occur, the experimental model used, the post‐seizure period, and the specific brain region analyzed. Two decades ago, initial studies investigating microglia in the developing brain following seizures focused primarily on rat models of status epilepticus (SE) induced by chemoconvulsants such as kainic acid (KA) and pilocarpine. In rats of nine postnatal days, no microglial changes were observed after SE.^67^, ^68^, ^69^, ^70^ However, at later postnatal days, SE induces morphological changes indicative of microglial activation.^67^, ^69^, ^70^, ^71^ By postnatal day 15 and into the third week of life, a marked microglial response to seizures is evident. These responses include morphological changes, such as an enlarged soma with shorter and thicker processes, and amoeboid shape, occurring as early as 2 h after SE onset. This activation persists, with an increased number of microglial cells detected 24 to 72 h after SE, as well as in the chronic period.^67^, ^68^, ^69^, ^70^, ^72^ Although most studies have focused on the hippocampus, particularly the CA1 and CA3 pyramidal layers and the DG granule cell layer, similar patterns have been observed in other brain regions, such as the basolateral nucleus of the amygdala and the dorsomedial nucleus of the thalamus.^71^, ^73^

The observed microgliosis following SE has been strongly linked to neurodegeneration, largely driven by pro‐inflammatory mechanisms.^67^, ^68^, ^69^, ^70^ However, previous studies often used semi‐quantitative analyses, lacked detailed descriptions of the stages of activation, and did not assess M1/M2 polarization. Despite these limitations, the studies consistently demonstrate the activation of microglia following SE. A stereology study showed a rapid microglial activation, characterized by a significant reduction in ramified microglia and an increase in hypertrophic microglia across all layers of CA1 and DG in 14‐day‐old rat pups submitted to lithium–pilocarpine SE. However, the total number of activated microglia, specifically bushy and amoeboid forms, only increased in regions where neurodegeneration was observed, such as the CA1 pyramidal layers and the DG granule cell layer and hilus. These results suggest that microglial activation following SE is closely associated with neuronal cell death in the hippocampus (unpublished data). Other studies indicated the importance of microglia for neuron loss and epileptogenesis, including its role in ferroptosis‐induced neuron death and in controlling neurogenesis, reinforcing the association between microglial activation and epileptogenesis.^74^, ^75^, ^76^, ^77^ Nevertheless, as we will discuss, depending upon microglial activation phenotype, these cells can be detrimental or protective.^78^

Recent studies have further clarified the role of microglial phenotypes after SE. Yang et al. demonstrated that the proportions of M1 and M2 microglia change after KA‐induced SE in 14‐day‐old mice.^79^ Using flow cytometry to identify cell surface markers, they found a high level of M1 microglia 7 days post‐SE, which subsided by day 14. M2 microglial levels also increased after SE, although to a lesser extent and for a shorter duration. Of interest, the altered M1/M2 balance was associated with damage to hippocampal dendrites 7 and 14 days after SE. Their study also showed that minocycline, a known inhibitor of microglial activation, reduced M1 microglia and promoted M2 polarization after SE, protecting against dendritic spine damage. Three days of minocycline treatment after SE also improved spatial learning and memory in immature mice by day 28 post‐SE. Based on these findings, the authors suggest that M1/M2 modulation in immature brain could be a promising target for seizure therapy.

Experiments conducted in zebrafish provide additional evidence of microglial activation in the developing brain following seizures. In 3 days post‐fertilization larvae, KA‐induced epileptiform activity increased the number of microglial cells 1 to 2 days post‐seizure. This response occurred alongside increased microglial phagocytosis of apoptotic nuclei, although the authors noted that microglia displayed non‐amoeboid morphologies, suggesting a different activation state. These changes were linked to increased seizure susceptibility in a pentylenetetrazol second‐hit model in developing brain.^80^ By using a genetic mutation in the developing zebrafish orthologue of the human *SCN1A* gene, associated with Dravet syndrome, the authors confirmed that mutant larvae had increased numbers of microglial cells and more apoptotic nuclei engulfed by microglia compared to controls.^80^ These findings support the active role of microglia in epilepsy in the developing brain.

Today it is widely accepted that microglia actively respond to SE in the developing brain and play a role in neurodegeneration following seizures. However, important questions remain, particularly regarding the role of microglia in epileptogenesis. It is essential to consider two key factors: the younger the brain, the less likely it is to develop epilepsy,^81^, ^82^ and most experimental approaches to studying epileptogenesis have focused on adult animals. For instance, in adult rats, 14 days of minocycline treatment following pilocarpine‐induced SE reduced the number, duration, and severity of spontaneous recurrent seizures (SRS), an effect attributed to the inhibition of microglial activation and pro‐inflammatory cytokine production in the hippocampus and cortex.^83^ However, some evidence challenges the antiepileptogenic effects of minocycline, as it did not prevent SRS after amygdala electrically induced SE, despite region‐selective neuroprotective effects.^84^ These findings highlight the importance of further research to clarify the role of microglia in epileptogenesis. It is also noteworthy that no similar studies have been conducted after SE in the developing brain.

**FIGURE 2:**
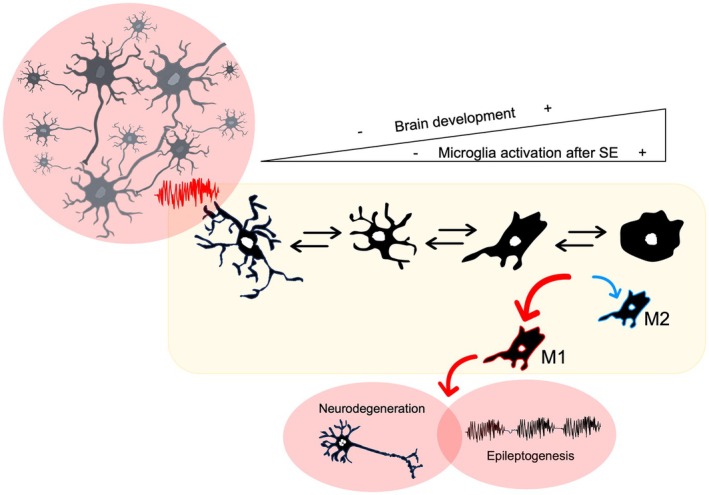
*Status epilepticus* (SE) promotes microglia activation in the immature brain, evidenced by an increase in microglial count, morphological changes, and the identification of M1 and M2 phenotypes. Current evidence supports microglial involvement in SE‐induced neurodegeneration and in epileptogenesis via several mechanisms, including via induction of neuron death, abnormal neurogenesis, and synapse reorganization, among others. Created using Microsoft PowerPoint for Mac v16.93.2.

Other studies aimed at understanding the role of microglia in seizures have focused on specific signal transduction pathways. For example, conditional *knockout* mice lacking apoptosis signal‐regulating kinase 1 (ASK1) in microglia submitted to KA‐induced SE present fewer pro‐inflammatory microglia, and reduced seizure frequency and neuron loss, suggesting that ASK1 inhibitors could offer neuroprotection against epilepsy.^85^ In addition, Peng and collaborators showed that rosiglitazone, a peroxisome proliferator‐activated receptor γ agonist, reversed the increase in M1 microglia after pilocarpine‐induced SE in adult mice, without affecting the latency to SE onset but improving overall outcomes.^86^ As a final remark to the importance of microglia in epileptogenesis, understanding better their morphological and biochemical dynamics and the timing of these changes presents an unparallel potential for new therapeutic interventions that target pro‐inflammatory processes and enhance neuroprotective mechanisms aimed at the epileptogenic window during brain development (Figure 2).

### 2.5 Participation of calpain in neuronal damage and neuroinflammation during epileptogenesis

#### 2.5.1 Calpains

Calpains are a family of calcium‐dependent cysteine proteases that regulate multiple biological processes that range from development to cell death.^87^, ^88^ In humans, this family comprises up to 15 different genes but the classical calpains, calpain‐1 and calpain‐2, are particularly abundant in the CNS. These two isoforms are the first described members of the family and carry out similar physiological functions; under resting conditions they reside in the cytosol as pro‐enzymes that are activated by autolysis upon calcium binding. The in vitro calcium levels required to activate calpain‐1 and calpain‐2 are different, since they require micromolar and millimolar calcium concentrations, respectively.^87^, ^88^

During postnatal development, the expression of calpain‐1 is upregulated, whereas calpain‐2 expression remains relatively constant.^89^ Concomitantly, calpain‐1 activity is highest during the prenatal period and decreases after birth before reaching adult levels, whereas calpain‐2 activity remains more or less constant during development in the hindbrain, but follows calpain‐1 decrease in the forebrain.^90^ Calpain activity is controlled by calpastatin, the only known endogenous proteinaceous inhibitor. During late pregnancy and early postnatal life calpastatin maintains low expression levels, allowing for a high calpain/calpastatin ratio that facilitates heightened calpain activation.^90^, ^91^ However, calpastatin expression almost doubles postnatally matching calpain‐1 expression changes and helping to prevent calpain overactivation in the adult brain.^89^, ^91^

#### 2.5.2 Calpain and neuronal death

Apoptotic neuronal death typically occurs in the immature brain but can also be provoked by noxious stimulation or injury as part of a process that can be morphologically differentiated from necrotic cell death.^92^ This process is to be differentiated from the one occurring in the adult brain where seizure‐induced neuronal death is morphologically necrotic but also triggers double‐stranded DNA cleavage with the characteristic “laddering” pattern. The differential morphology and the lack of caspase activation, which occurs only in the immature brain, points to neuronal necrosis as a main process linked to neuronal death induced by SE.^92^ The mechanistic origin of neuronal necrosis due to an acute neuronal injury appears to be the result of excessive *N*‐methyl‐d‐aspartate (NMDA) receptor activity. Overstimulation and opening of this type of receptor allows the entrance and accumulation of massive amounts of calcium ions that fuel calpain activation. Under physiological conditions, controlled calpain activation involves the activation of a few calpain molecules that perform controlled proteolysis, whereas sustained calcium overload under pathological conditions promotes a more generalized activation leading to uncontrolled proteolysis.^93^

Calpain overactivation is implicated in a wide range of neurotoxic states, which include stroke, traumatic brain injury, neurodegenerative disorders, and epilepsy. During prolonged seizures, excessive glutamate release overactivates glutamatergic receptors, promoting increased calcium influx, which, in turn, leads to calpain hyperactivation and neurotoxicity.^94^ Calpain dysregulation is observed following an episode of SE.^94^ More importantly, brain tissue resected from patients with epilepsy shows increased expression of calpain‐1 and calpain‐2, and studies with animal models have linked increased calpain‐2 expression with neurodegeneration.^95^, ^96^

Proteolytic processing is critical for excitotoxicity, but the identity of most cleaved proteins is unknown.^97^ Calpain‐dependent proteolysis of intracellular substrates leads to disruption of the structural integrity and functional activity of neurons. Pilocarpine‐induced SE leads to sustained removal of γ‐aminobutyric acid A receptor (GABA_A_R) from the plasma membrane and reduces receptor expression, effectively inactivating them.^98^ In addition to receptor loss, other proteins key for inhibitory neurotransmission like gephyrin, VGAT, and KCC2 are also downregulated.^99^, ^100^ These proteins required for normal inhibitory drive undergo increased cleavage by calpain, suggesting that calpain‐mediated proteolysis plays a key role in the loss of inhibitory neurotransmission.^99^, ^100^ During brain development, SE does not induce significant neuron loss or increase in calpain‐related proteolytic products in the early postnatal period (i.e., days 7 and 14), whereas by day 21 the animals have a similar response to adults.^101^, ^102^ In a model of epileptic spasms, pre‐treatment with calpain‐2 inhibition before NMDA stimulation reduces frequency and increases latency to spasms, and reverses abnormalities in GABAergic neurotransmission.^103^ As calpain‐2 is proposed to be detrimental while calpain‐1 is neuroprotective,^95^, ^104^ calpain‐2‐dependent proteolysis is a relevant mechanism behind SE‐induced neuron loss in rats older than 14 days, but not in newborns, and other mechanisms associated with epilepsy are calpain dependent.

#### 2.5.3 Calpain and inflammation

Inflammation is a key mediator of cerebral damage due to seizures and epilepsy. Besides playing a role in neurotoxic events, calpains are involved in both innate and adaptive immunity. Epileptic tissue from human and animal models contains increased number of cells from the innate immune response, and the levels of activated monocytes and macrophages directly correlates with seizure activity and BBB disruption.^105^ In fact, recurrent seizures are powerful inducers of brain inflammation that boost monocyte recruitment and promote astrocyte and microglia activation.^50^, ^105^ Calpains are expressed and regulate immunological functions of many cell types in the immune system including macrophages, neutrophils, and lymphocytes.^106^, ^107^ Pharmacological inhibition of calpain attenuates acute and chronic inflammation, preserves BBB integrity and, as a secondary effect, reduces neutrophil recruitment.^108^, ^109^, ^110^

In addition, seizures affect the expression of a wide variety of inflammatory factors including interleukin (IL)‐1β, chemokines, complement, and other immune regulatory molecules. Altered expression of classic inflammatory cytokines, as well as HMGB1, might contribute to the pathogenesis of epilepsy.^111^, ^112^ Moreover, increased levels of classical proinflammatory cytokines IL‐1β, IL‐6, and tumor necrosis factor (TNF)‐α can be detected in peripheral blood after recent seizure activity.^113^ These molecules, in turn, also promote excitability. For instance, IL‐1β‐mediated activation of interleukin receptor initiates a cascade of downstream effectors that disrupt neuronal networks and produce hyperexcitability.^111^

Calpains are relevant for both the recruitment and activation of inflammatory cells, for the secretion of several inflammatory cytokines, and also mediate the cleavage of pre‐interleukins, increasing their affinity to interleukin receptors.^109^, ^114^ Calpain activation may also contribute to the amplification of inflammatory responses by facilitating the nuclear localization of interleukins. In addition, the production and release of prostaglandins by injured neurons can exacerbate inflammation and also impact seizure control by antiseizure medication, as these prostaglandins induce P‐glycoprotein, a transporter protein associated with drug resistance expressed in blood vessels.^115^, ^116^, ^117^ In vitro calpain treatment was shown to increase P‐glycoprotein proteolysis, indicating calpains can also impact drug resistance in patients with epilepsy.^118^ Thus calpains are involved not only in the inflammation response but they can affect drug responsiveness in epilepsy, and thus they may serve as molecular targets to develop immunomodulatory drugs for the exaggerated activation of inflammasomes and production of pro‐inflammatory cytokines responsible for tissue and organ damage.

## 3 CONCLUSIONS

The existence of a close relationship between the different organs and systems through the neuroimmune axis shows the importance of the delicate balance of the immature immune system during neurodevelopment, which due to its heterogeneous dynamics in each critical period of development, when tested by the various challenges of the postnatal environment, is highly susceptible to long‐term damage and alterations mediated by the imbalance in the modulation of the inflammatory response. We know that neuroinflammation can favor the development and evolution of neurological disorders such as seizures and epilepsy progression; however, we still do not fully understand the direction and evolution of the patterns of damage generated in the postnatal stage until they are symptomatic and visible in later infant or adult stages. In addition, structural damage and the evident molecular alterations are dependent on the neurodevelopmental window in which they are generated.

Therefore, it is vitally important to generate a better understanding of this neuroimmune axis and to be able to establish an appropriate therapeutic approach at each stage of life. This exposes the need for treatment strategies that help the immature immune system to modulate the inflammatory response and neuronal hyperexcitability without long‐term alterations in the neuronal networks. A possible strategy would be to promote the activation of endogenous anti‐inflammatory and antioxidant mechanisms that help recover a normal neuroimmune state, for which the development and monitoring of immature experimental models and their evaluation in later or adult stages is important, which will allow us to better understand this neuroimmune axis and its evolution during epileptogenesis.

## AUTHOR CONTRIBUTIONS

*Literature review; data curation; writing and reviewing original draft*: Angelica Vega García, María Leonor López‐Meraz, and Marco I. González. *Conceptualization; reviewing original draft; reviewing and editing; supervision*: Luisa Rocha. *Literature review; data curation; writing and reviewing original draft; editing*: Jose Eduardo Peixoto‐Santos. *Conceptualization; literature review; data curation; writing and reviewing original draft; editing; supervision*: Esper Abrão Cavalheiro.

## ETHICAL STATEMENT

We confirm that we have read the Journal's position on issues involved in ethical publication and affirm that this report is consistent with those guidelines.

## Data Availability

Data sharing not applicable to this article as no datasets were generated or analysed during the current study.
